# Anisotropic Optical and Vibrational Properties of GeS

**DOI:** 10.3390/nano11113109

**Published:** 2021-11-18

**Authors:** Natalia Zawadzka, Łucja Kipczak, Tomasz Woźniak, Katarzyna Olkowska-Pucko, Magdalena Grzeszczyk, Adam Babiński, Maciej R. Molas

**Affiliations:** 1 Institute of Experimental Physics, Faculty of Physics, University of Warsaw, 02-093 Warsaw, Poland; n.zawadzka2@student.uw.edu.pl (N.Z.); lucja.kipczak@fuw.edu.pl (Ł.K.); k.olkowskapu@student.uw.edu.pl (K.O.-P.); Magdalena.Grzeszczyk@fuw.edu.pl (M.G.); adam.babinski@fuw.edu.pl (A.B.); 2 Department of Semiconductor Materials Engineering, Wrocław University of Science and Technology, 50-370 Wrocław, Poland; tomasz.wozniak@pwr.edu.pl

**Keywords:** monochalcogenides, germanium sulfide, photoluminescence, reflectance contrast, excitons, Raman scattering, Density Functional Theory, phonons

## Abstract

The optical response of bulk germanium sulfide (GeS) is investigated systematically using different polarization-resolved experimental techniques, such as photoluminescence (PL), reflectance contrast (RC), and Raman scattering (RS). It is shown that while the low-temperature (*T* = 5 K) optical band-gap absorption is governed by a single resonance related to the neutral exciton, the corresponding emission is dominated by the disorder/impurity- and/or phonon-assisted recombination processes. Both the RC and PL spectra are found to be linearly polarized along the armchair direction. The measured RS spectra over a broad range from 5 to 300 K consist of six Raman peaks identified with the help of Density Functional Theory (DFT) calculations: A${}_{g}^{1}$, A${}_{g}^{2}$, A${}_{g}^{3}$, A${}_{g}^{4}$, B${}_{1g}^{1}$, and B${}_{1g}^{2}$, which polarization properties are studied under four different excitation energies. We found that the polarization orientations of the A${}_{g}^{2}$ and A${}_{g}^{4}$ modes under specific excitation energy can be useful tools to determine the GeS crystallographic directions: armchair and zigzag.

## 1. Introduction

Two-dimensional (2D) layered van der Waals (vdW) semiconductors, such as transition metal dichalcogenides (e.g., MoS${}_{2}$ and WSe${}_{2}$) and post-transition metal dichalcogenides (e.g., InSe), have appeared as a fascinating class of materials for exploring novel excitonic phenomena [1,2,3,4,5]. In terms of their crystal structures, these materials are characterized by a high in-plane symmetry. On the other hand, there is a group of materials with a low in-plane symmetry, which includes, e.g., black phosphorus (BP) [6,7,8], or Re-based dichalcogenides (ReS${}_{2}$ and ReSe${}_{2}$ [9,10]. Among these anisotropic materials, a new group of emerging vdW semiconductors, i.e., group-IV monochalcogenides MX (where M = Ge, Sn, or Pb and X = S, Se, or Te), has attracted increasing attention due to their anisotropic optical properties. They originate from a low-symmetry orthorhombic crystal structure, analogous to BP. Moreover, the family of MX materials exhibits high carrier mobility, larger for monolayers as compared to bulk [11], which can lead to potential applications in angle-resolved opto-electronics.

Despite the fact that the properties of GeS have been studied in several papers [12,13,14,15,16,17], most of these investigations were limited to a single particular experimental technique, e.g., photoluminescence (PL). In this work, we study anisotropic optical and vibrational properties of GeS with the aid of three types of optical experiments, moreover, performed with polarization resolution. The techniques of choice are PL, reflectance contrast (RC), and Raman scattering (RS), which are complement one another. While the PL and RC experiments give us correspondingly access to occurring emission and absorption processes, the RS measurements provide the information about Raman-active phonons. We found that the low-temperature (*T* = 5 K) optical band-gap absorption is governed by a single resonance related to the neutral exciton, while the corresponding emission is dominated by disorder/impurity- and/or phonon-assisted recombination processes. Moreover, both the RC and PL spectra are found to be linearly polarized along the armchair direction of the crystal, which is consistent with previous works [12,16,17]. The measured RS spectra as a function of temperature from 5 to 300 K are composed of six Raman peaks, the energies and symmetries of which are in agreement with phonon dispersion calculated using Density Functional Theory (DFT). Note that the only four high-energy phonon modes have been reported so far [12,13,14,15]. Moreover, the polarization properties of the measured RS peaks are studied under four different excitation energies. We found that the polarization orientations of the A${}_{g}^{2}$ and A${}_{g}^{4}$ modes under specific excitation energy can be used to distinguish between armchair and zigzag crystallographic directions in GeS crystals.

## 2. Materials and Methods

### 2.1. Samples

A bulk-like flake of GeS was placed on a Si/(90 nm) SiO${}_{2}$ substrate by polydimethylsiloxane (PDMS)-based exfoliation [18] of bulk crystals purchased from HQ Graphene. The PDMS stamp was prepared from the gel-film purchased from Gel-Pak. The flake of interest was initially identified by visual inspection under an optical microscope then subjected to atomic force microscopy.

### 2.2. Experimental Techniques

The PL spectra measured under laser excitation of λ = 660 nm (1.88 eV). The RS measurements were performed using illumination with a series of lasers: λ = 488 nm (2.54 eV), λ = 515 nm (2.41 eV), λ = 561 nm (2.21 eV), λ = 633 nm (1.96 eV). The excitation light in those experiments was focused by means of a 50× long-working distance objective with a 0.55 numerical aperture (NA) producing a spot of about 1 μm diameter. The signal was collected via the same microscope objective (the backscattering geometry), sent through a 0.75 m monochromator, and then detected by using a liquid nitrogen cooled charge-coupled device (CCD) camera. To detect low-energy RS below 100 cm${}^{-1}$ from the laser line, a set of Bragg filters was implemented in both excitation and detection paths. In the case of the RC studies, the only difference in the experimental setup with respect to the one used for recording the PL and RS signals concerned the excitation source, which was replaced by a tungsten halogen lamp. The light from the lamp was coupled to a multimode fiber of a 50 μm core diameter, and then collimated and focused on the sample to a spot of approximately 4 μm diameter. All measurements were performed with the samples placed on a cold finger in a continuous flow cryostat mounted on x–y manual positioners. The excitation power focused on the sample was kept at 50 μW during all measurements to avoid local heating.

The polarization-resolved PL and RC spectra were analyzed by the motorized half-wave plate and a fixed linear polarizer mounted in the detection path. In contrast, the polarization-sensitive RS measurements were performed in two co- (XX) and cross-linear (XY) configurations, which correspond to the parallel and perpendicular orientation of the excitation and detection polarization axes, respectively. The analysis of the RS signal was done using a motorized half-wave plate, mounted on top of the microscope objective, which provides simultaneous rotation of polarization axis in the XX and XY configurations.

### 2.3. Theoretical Calculations

DFT calculations were performed in Vienna Ab initio Simulation Package [19] with Projector Augmented Wave method [20] and Perdew–Burke–Ernzerhof parametrization [21] of general gradients approximation of the exchange-correlation functional. A plane wave basis energy cutoff of 550 eV and a Γ-centered Monkhorst–Pack k-grid of 12 × 10 × 4 were found sufficient to converge the lattice constants up to 0.001 Å. Geometrical parameters were optimized until the interatomic forces and stress tensor components were lower than 10${}^{-5}$ eV/Å and 0.01 kbar, respectively. The interlayer vdW interactions were taken into account by the use of Grimme’s D3 correction [22]. Phonon dispersion of GeS was calculated within Parliński–Li–Kawazoe method [23], as implemented in Phonopy software [24]. The 4 × 4 × 2 supercells were employed to find the interatomic force constants within the harmonic approximation. The irreducible representations of Raman active phonon modes at Γ point were determined with the use of spglib library [25].

## 3. Results

### 3.1. Crystallographic Structure

**Figure 1:**
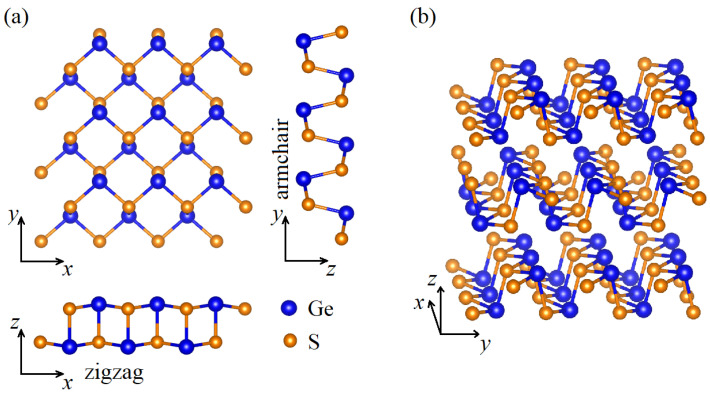
(**a**) Top and side views of the GeS crystal structure for a single layer. The armchair and zigzag directions are shown in relation to the crystal orientation and lattice parameters. (**b**) Geometrical structure of multilayer GeS.

GeS is a layered material, which crystallizes in a distorted orthorhombic structure (D${}^{1}6_{2h}$), as shown in Figure 1. That form, comprising eight atoms in the primitive unit cell, has been proven to be dynamically and thermally stable at room temperature [26]. The puckered honeycomb lattice of GeS has an anisotropic crystal structure characterized by the two orthogonal armchair (*x*) and zigzag (*y*) directions, denoted in Figure 1a. The stack of consecutive layers in the perpendicular direction (*z*) in respect to the xy plane is presented in Figure 1b. Note that the crystallographic structure of GeS is analogous to the BP one [8].

### 3.2. Optical Properties

**Figure 2:**
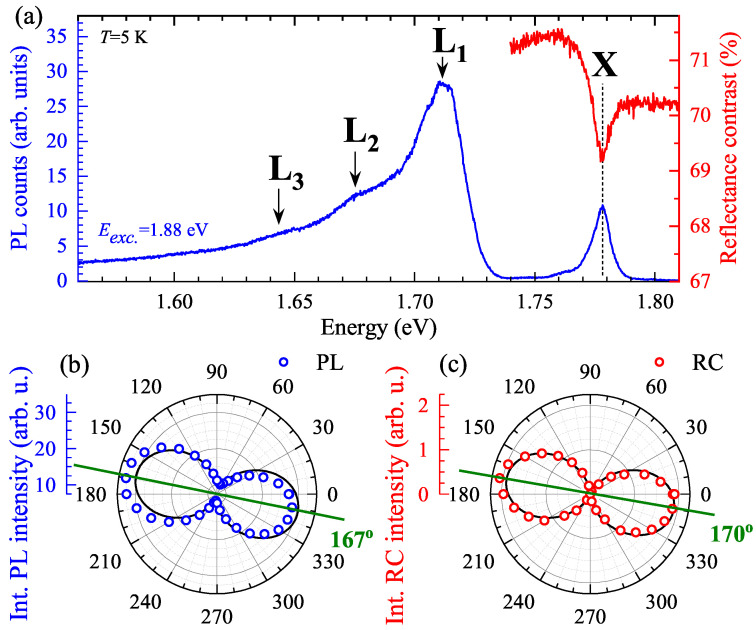
(**a**) The low-temperature (*T* = 5 K) PL (blue curve) and RC (red curve) spectra measured on the GeS flake. Polar plots of the integrated intensities of the X transitions from the (**b**) PL and (**c**) RC spectra.

Figure 2a presents the low-temperature (*T* = 5 K) PL and RC spectra. The PL spectrum consists of four emission lines, denoted as X, L${}_{1}$, L${}_{2}$, and L${}_{3}$. In contrast, the corresponding RC spectrum consists of a single resonance, which energy coincides with the X emission line. To examine the origin of the X transition, we carried out the polarization resolved measurements of PL and RC spectra. The extracted polarization dependencies of the X transitions are presented in Figure 2b,c. Solid lines in the Figs represent fits of the evolution of the PL/RC intensity as a function of light polarization. A dichroic relation of polarized light using Malus law was used: I(θ): [27]

(1) $$I\left(\theta \right)=Acos^{2}\left(\theta -\phi \right),$$

where *A* is the amplitude of the emission/absorption transition and ϕ represents the phase of polarization dependence. It is seen that both the emission and absorption signals of the X transitions are linearly polarized along the same direction (167° and 170°, respectively). The result confirms directly the same origin of the X feature apparent in both the PL and RC spectra, as previously reported independently for the emission [12], photoreflectance [16], or absorption [17]. Moreover, according to the authors of [17], the X resonance can be related to the direct transition at the Γ point of the Brillouin zone (BZ), which is linearly polarized along the armchair crystallographic direction. Using DFT methods, we also calculated the band-gap energy at the Γ point of the BZ, which is on the order of 1.749 eV. The obtained value is smaller than the energy of the X transition (∼1.78 eV), which is probably due to underestimation of the band-gap energy with DFT calculations.

**Figure 3:**
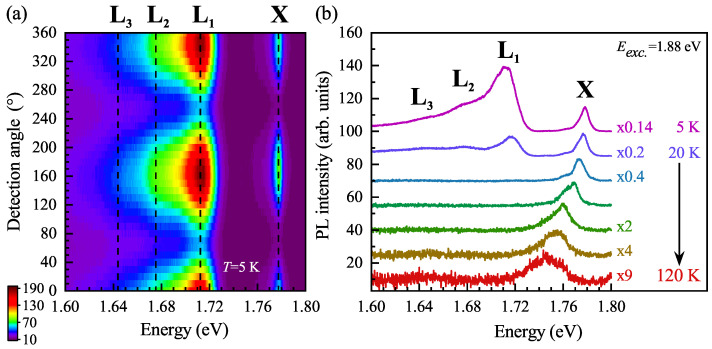
(**a**) False-color map of low-temperature PL spectra of GeS as a function of the detection angle of linear polarization under 1.88 eV laser light excitation. (**b**) Temperature evolution of the PL spectra measured on GeS. The spectra are vertically shifted for clarity and some of them are multiplied by scaling factors for more clarity.

In order to study the origin of L lines, the polarization-resolved and temperature evolution of the PL spectra was measured, see Figure 3a,b. As can be appreciated in the Figure 3a, the L${}_{1}$, L${}_{2}$, and L${}_{3}$ lines are linearly polarized along the same armchair direction as the neutral exciton transition. Moreover, with increasing temperature, the low-energy L${}_{1}$, L${}_{2}$, and L${}_{3}$ peaks quickly disappear from the spectra. At *T* = 40 K, only the neutral exciton contributes to the PL spectrum. The further increase of temperature leads to the typical redshift and the linewidth broadening of the neutral exciton, which can be observed up to 120 K. The observed temperature dependence of the L lines is very similar to the previously reported behavior of so-called “localized” excitons in monolayers of WS${}_{2}$ and WSe${}_{2}$ exfoliated on Si/SiO${}_{2}$ substrates [2,4,28,29,30,31]. Consequently, we can ascribe tentatively the L${}_{1}$, L${}_{2}$, and L${}_{3}$ peaks to disorder/impurity- and/or phonon-assisted recombination processes.

### 3.3. Vibrational Properties

**Figure 4:**
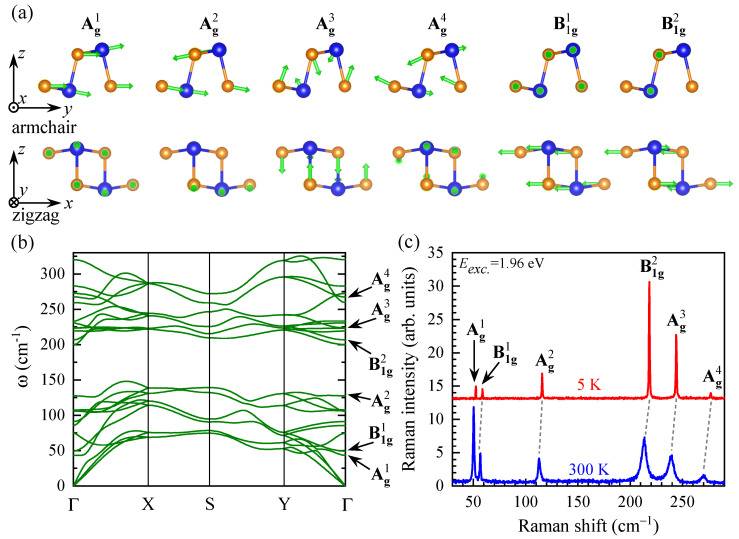
(**a**) Atom displacements (green arrows) for the Raman-active modes. Axes indicate two view perspectives: armchair and zigzag directions. (**b**) The calculated phonon dispersion of GeS. (**c**) RS spectra measured on GeS at low (*T* = 5 K) and room (*T* = 300 K) temperatures. The spectra are vertically shifted for clarity.

As GeS belongs to the point group D${}_{2h}$, there are 12 Raman-active modes: 4A${}_{g}$, 2B${}_{1g}$, 2B${}_{2g}$, and 4B${}_{3g}$. According to the polarization selection rules for the space group Pnma (No. 62) of GeS, four A${}_{g}$ and two B${}_{1g}$ phonon modes should be observed in backscattering geometry along the *z* crystallographic direction. Their corresponding atomic displacements, denoted by green arrows, are presented in Figure 4a. The modes are classified according to their irreducible representations in the point group D${}_{2h}$, and additionally numbered due to their increased Raman shift (top index). As can be seen in the Figure 4a, the A${}_{g}$ modes presents atom movement mostly in the plane defined by the armchair and out-of-plane directions, while the B${}_{1g}$ vibrations take place along zigzag direction. Figure 4b presents the calculated phonon dispersion with marked A${}_{g}$ and B${}_{1g}$ phonon modes active in our experimental conditions. To verify the theoretical calculations, we measured the RS spectra of GeS at low (*T* = 5 K) and room (*T* = 300 K) temperatures. Both the RS spectra consist of six Raman modes: A${}_{g}^{1}$, A${}_{g}^{2}$, A${}_{g}^{3}$, A${}_{g}^{4}$, B${}_{1g}^{1}$, and B${}_{1g}^{2}$, which energies are in good agreement with theoretical calculations shown in Figure 4b. Note that the assignments of Raman peaks in GeS vary significantly in previous reports [12,13,14,15]. For example, the B${}_{1g}^{2}$ peak, has been attributed to the phonons of different symmetries, i.e., B${}_{3g}$ in [12,13,14] and B${}_{1g}$ in [15]. However, only phonon modes of the B${}_{1g}$ symmetry can be observed in RS spectra measured in backscattering geometry along the *z* crystallographic direction. Moreover, the characteristic effect of temperature on the observed peaks can be appreciated, which is analysed in Appendix A. Particularly, it is seen that both the redshift and the broadening of Raman peaks scales with the increasing phonon energy, e.g., the temperature-induced change of the energy and linewidth of the A${}_{g}^{1}$ mode is much smaller as compared to the A${}_{g}^{3}$.

**Figure 5:**
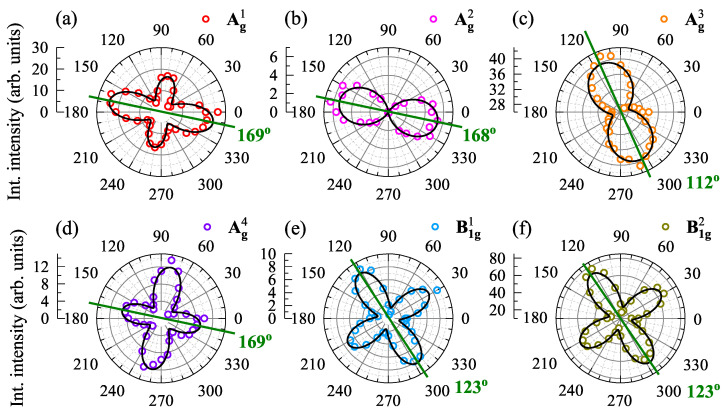
Polar plots of the integrated intensities of the phonon modes: (**a**) A${}_{g}^{1}$, (**b**) A${}_{g}^{2}$, (**c**) A${}_{g}^{3}$, (**d**) A${}_{g}^{4}$, (**e**) B${}_{g}^{1}$, and (**f**) B${}_{g}^{2}$, measured on GeS at *T* = 300 K under 1.96 eV excitation. The green lines on polar plots are along polarization axes of modes.

To verify our attribution of the observed Raman peaks, we measured the polarization-resolved RS spectra on GeS at *T* = 300 K under 1.96 eV excitation. Figure 5 presents polar plots of the integrated intensities as a function of detection angle for all observed phonon modes in co-linear configuration (XX). As the corresponding results in cross-linear configuration (XY) do not add additional value, we focused on XX configuration in our analysis [7]. Solid lines represent fits of the modes intensities as a function of light polarization, I(θ), described by [7]

(2) $$I\left(\theta \right)=(|a|sin^{2}\left(\theta -\phi \right)+|c|cos\xi cos^{2}{\left(\theta -\phi \right))}^{2}+{\left|c\right|}^{2}sin^{2}\xi cos^{4}\left(\theta -\phi \right),$$

where |a| and |c| are the amplitudes of the phonon modes, ϕ represents the phase of polarization dependence, ξ represents the phase difference. It is seen that the polarization axes of the A${}_{g}^{1}$, A${}_{g}^{2}$, and A${}_{g}^{4}$ modes, marked by green lines in the Figure 5, are approximately oriented in the same direction, i.e., 169°, 168°, and 169°, respectively. The direction is the same as the orientation apparent in PL and RC spectra (167° and 170°, respectively), which corresponds to the armchair crystallographic direction. The A${}_{g}^{3}$ mode exhibits different polarization axis of about 112°, which does not match any crystallographic direction. In contrast, the polarization axes of the B${}_{1g}^{1}$ and B${}_{1g}^{2}$ point to the same direction (123°), which is shifted of about 45° from the crystallographic directions. In terms of observed symmetries of phonon modes, the A${}_{g}^{2}$ and A${}_{g}^{3}$ modes display 2-fold symmetry with an angle period of 180°. In contrast, the A${}_{g}^{1/4}$ (B${}_{1g}^{1/2}$) mode presents the 4-fold symmetry with an angle period of 90° and with the different (the same) intensity of perpendicular arms. Consequently, the polarization axes of the other phonon modes (except the A${}_{g}^{3}$) can be used to determine the crystallographic direction, but without the discrimination between the zigzag and armchair directions. Only the 2-fold symmetry of the A${}_{g}^{2}$ mode allows to determine the armchair direction of the crystal.

**Figure 6:**
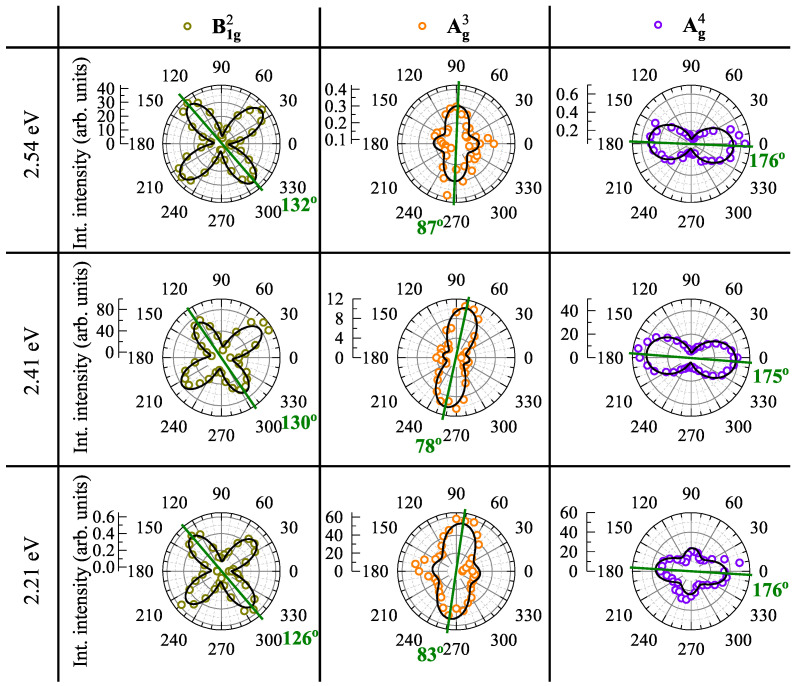
Polar plots of the integrated intensities of the phonon modes B${}_{1g}^{2}$, A${}_{g}^{3}$, and A${}_{g}^{4}$ measured on GeS at *T* = 300 K under 2.54 eV, 2.41 eV, and 2.21 eV excitation. The green lines on polar plots are along polarization axes of modes.

In order to examine the effect of the excitation energy on the polarization properties of phonon modes, we performed the polarization-resolved RS experiments under three more different excitations (2.21 eV, 2.41 eV, and 2.54 eV). Due to our experimental limitations, the polarization properties of only three modes, i.e., B${}_{1g}^{2}$, A${}_{g}^{3}$, and A${}_{g}^{4}$, were analyzed, see Figure 6. Three polarization characteristics of phonon modes can be distinguished: (i) The B${}_{g}^{1}$ mode conserves the polarization axis and shape under excitation with different laser wavelength. (ii) The A${}_{g}^{3}$ mode dramatically changes its polarization axis (compare Figure 5 and Figure 6). Moreover, its symmetry also changes: from 2-fold symmetry under 1.96 eV excitation to 4-fold symmetry under other excitations. (iii) In contrast, the 4-fold symmetry of the A${}_{g}^{4}$ mode under 1.96 eV excitation changes gradually to the 2-fold one under 2.41 eV and 2.54 eV excitations. Its 2-fold symmetry can be useful to determine crystallographic direction of the crystal. One can conclude that the different excitation energies may affect significantly the shape between 2- and 4-fold of the A${}_{g}^{3}$ and A${}_{g}^{4}$ modes.

**Figure 7:**
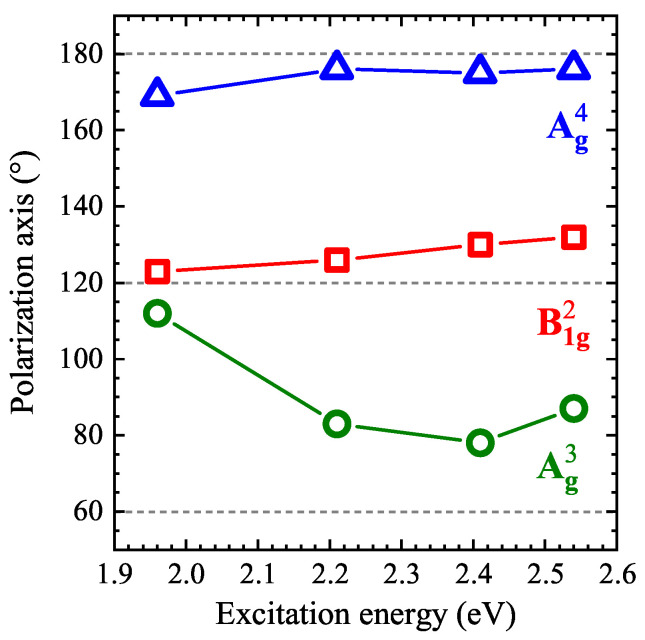
The effect of the excitation energy on the polarization axes of three phonon modes: B${}_{1g}^{2}$, A${}_{g}^{3}$, and A${}_{g}^{4}$.

The influence of the excitation energies on the polarization axes of three investigated phonon modes, i.e., B${}_{1g}^{2}$, A${}_{g}^{3}$, and A${}_{g}^{4}$, is summarized in Figure 7. It can be seen that the polarization axes of the B${}_{1g}^{2}$ and A${}_{g}^{4}$ modes do not change significantly as a function of excitation energy, whereas the difference between the polarization axes of the A${}_{g}^{3}$ mode increases of about 34° between the 1.93 eV and 2.41 eV excitations. Note that the previous works devoted to the polarization properties on Raman peaks were limited [12,13,15]. Particularly, the reported polarization-resolved RS spectra were detected for Raman shifts larger than ~100 cm${}^{-1}$ [12,13,15], using two specific excitations, i.e., 1.96 eV [12,15] and 2.33 eV [13], and additionally the RS spectra were measured only in given crystallographic directions (armchair and zigzag) [12,15]. Nevertheless, our results are consistent with the ones reported in [12,13,15]. Due to the observed behavior of different peaks, we can assume that (i) the polarization properties of the B${}_{1g}^{2}$ mode can be used to determine the crystallographic axes in GeS, but without attribution the zigzag and armchair directions; (ii) the variation of the polarization axis of the A${}_{g}^{3}$ as a function of the excitation energy suggests that the electron-phonon coupling may change the A${}_{g}^{3}$ polarization axis (see the work in [32] for details); and (iii) due to 2-fold symmetries of the polarization properties (except for 1.96 eV) and the almost fixed polarization axis of the A${}_{g}^{4}$ mode, its polarization properties are good to identify the zigzag and armchair crystallographic directions.

Note that the observed influence of the excitation energies on the axes and shape of polarization properties of phonon modes is very similar to those reported for different anisotropic layered materials, e.g., BP, ReS${}_{2}$, ReSe${}_{2}$, SnSe${}_{1-x}$S${}_{x}$ [6,9,32,33,34]. Moreover, as the effect of thickness on the polarization properties of different modes in BP has been reported [6,33], the outline of further research on thin layers of GeS seems to be clear.

## 4. Conclusions

We have presented systematic studies of the optical and vibrational properties of GeS. It has been found that while the low-temperature (*T* = 5 K) optical band-gap absorption is governed by a single resonance related to the neutral exciton, the corresponding emission is dominated by the disorder/impurity- and/or phonon-assisted recombination processes. Moreover, both the RC and PL spectra are found to be linearly polarized along the armchair crystallographic direction. We propose using the effect to determine crystallographic direction of GeS. The effect of the excitation energy on the polarization properties of different phonon modes has been analyzed. It has been shown that the polarization orientation of the A${}_{g}^{2}$ and A${}_{g}^{4}$ modes under specific excitation energy can be useful tools to determine the GeS crystallographic directions: armchair and zigzag. The strong dependence of the A${}_{g}^{3}$ mode polarization on the excitation light energy strongly suggests its strong coupling to electronic excitation of the crystal. We believe that the observations will trigger more theoretical studies to explain the origin of the electron–phonon interaction.

## Figures and Tables

**Figure A1:**
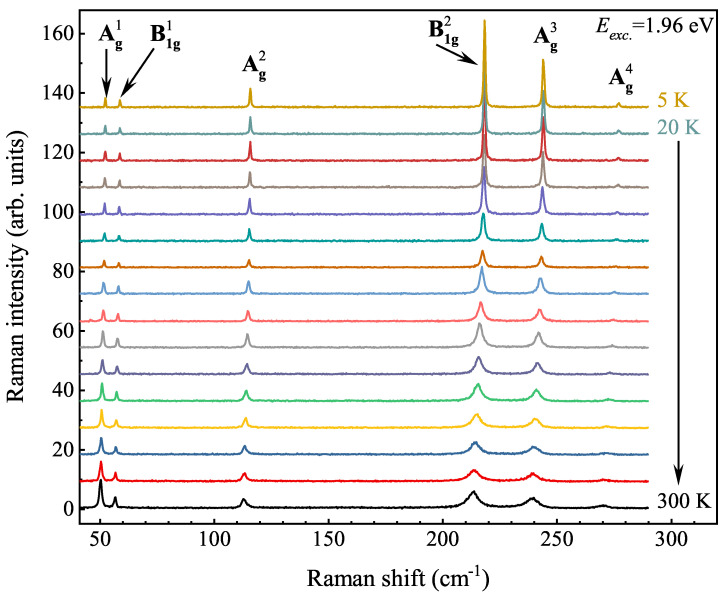
Raman scattering spectra measured on GeS as a function of temperature in range from 5 K to 300 K under 1.96 eV excitation.

**Figure A2:**
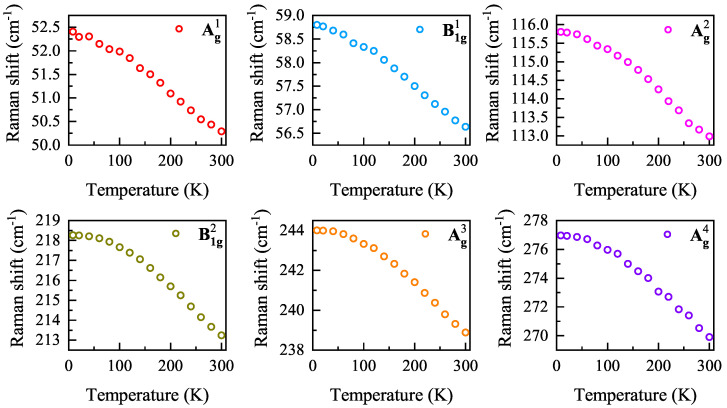
Temperature evolution of the Raman shifts for the all observed phonon modes.

**Figure A3:**
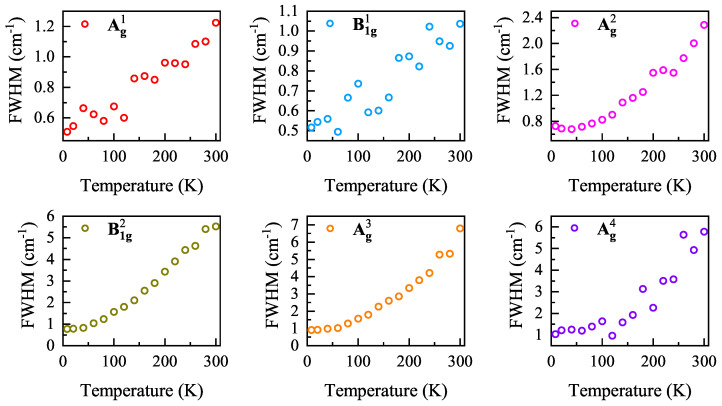
Temperature evolution of the FWHMs for the all observed phonon modes.

## Data Availability

The data presented in this study are available on request from the corresponding author.

## Appendix A. The Temperature Dependence of the Raman Scattering Spectra

Figure A1 presents the unpolarized Raman scattering spectra of GeS measured as a function of temperature over a broad range from 5 to 300 K. As can be appreciated in the Figure, all RS spectra consist of six peaks: A${}_{g}^{1}$, A${}_{g}^{2}$, A${}_{g}^{3}$, A${}_{g}^{4}$, B${}_{1g}^{1}$, and B${}_{1g}^{2}$. Moreover, the effect of temperature on their energies and full widths at half maximum (FWHMs) can be seen. In order to study this effect, we fitted the observed phonon modes. Figure A2 and Figure A3 show the temperature dependencies of the Raman shifts and FWHMs for all the observed Raman peaks, respectively. The temperature evolutions of the phonon energies experience typical redshifts with increased temperature. Moreover, it can be seen that the total redshifts grow with the increased phonon energies. It starts from approximately 2–3 cm${}^{-1}$ for A${}_{g}^{1}$, B${}_{1g}^{1}$, and A${}_{g}^{2}$ modes, through ∼5 cm${}^{-1}$ for the B${}_{1g}^{2}$ and A${}_{g}^{3}$ modes, ending with around 7 cm${}^{-1}$ for the A${}_{g}^{4}$ mode. The analogous effect of phonon energies is also apparent for the temperature evolution of the FWHMS for phonon modes, see Figure A3. The FWHMs grow of about two times for A${}_{g}^{1}$ and B${}_{1g}^{1}$ modes is transition from 5 to 300 K, through three times for the A${}_{g}^{2}$ mode, and finally reaches 6–7 times increase for the B${}_{1g}^{2}$, A${}_{g}^{3}$, and A${}_{g}^{4}$ modes. The observed effect of phonon energies on their temperature evolutions of energies and linewidths is very interesting and requires sophisticated theoretical analysis, which stays out of the scope of this work.
